# Cortical output to fast and slow muscles of the ankle in the rhesus macaque

**DOI:** 10.3389/fncir.2013.00033

**Published:** 2013-03-01

**Authors:** Heather M. Hudson, Darcy M. Griffin, Abderraouf Belhaj-Saïf, Paul D. Cheney

**Affiliations:** Department of Molecular and Integrative Physiology, University of Kansas Medical Center, Kansas CityKS, USA

**Keywords:** soleus, tibialis anterior, cortical facilitation, EMG, stimulus triggered averaging

## Abstract

The cortical control of fast and slow muscles of the ankle has been the subject of numerous reports yielding conflicting results. Although it is generally agreed that cortical stimulation yields short latency facilitation of fast muscles, the effects on the slow muscle, soleus, remain controversial. Some studies have shown predominant facilitation of soleus from the cortex while others have provided evidence of differential control in which soleus is predominantly inhibited from the cortex. The objective of this study was to investigate the cortical control of fast and slow muscles of the ankle using stimulus triggered averaging (StTA) of EMG activity, which is a sensitive method of detecting output effects on muscle activity. This method also has relatively high spatial resolution and can be applied in awake, behaving subjects. Two rhesus macaques were trained to perform a hindlimb push-pull task. Stimulus triggered averages (StTAs) of EMG activity (15, 30, and 60 μA at 15 Hz) were computed for four muscles of the ankle [tibialis anterior (TA), medial gastrocnemius (MG), lateral gastrocnemius (LG), and soleus] as the monkeys performed the task. Poststimulus facilitation (PStF) was observed in both the fast muscles (TA, MG, and LG) as well as the slow muscle (soleus) and was as common and as strong in soleus as in the fast muscles. However, while poststimulus suppression (PStS) was observed in all muscles, it was more common in the slow muscle compared to the fast muscles and was as common as facilitation at low stimulus intensities. Overall, our results demonstrate that cortical facilitation of soleus has an organization that is very similar to that of the fast ankle muscles. However, cortical inhibition is organized differently allowing for more prominent suppression of soleus motoneurons.

## Introduction

The existence of fast and slow motor units is well known (Eccles et al., 1958; Andersen and Sears, 1964; Kugelberg and Edstrom, 1968; Ranvier, 1874; Kronecker and Stirling, 1878). The ankle muscles have been a particular focus of many studies investigating the distribution, metabolism, and physiology of fast and slow motor units. This work has established that the soleus muscle consists exclusively of slow motor units while tibialis anterior (TA), an ankle flexor, consists largely of fast motor units. MG and LG are mixed but with a predominance of fast motor units (Burke, 1967; Burke et al., 1970, 1971; Burke and Tsairis, 1974). The hypothesis of differential cortical control of these exclusively or predominantly fast and slow muscles has been the subject of numerous studies in cats, primates and humans yielding conflicting results.

Monosynaptic linkages have been established between corticospinal neurons and hindlimb motoneurons in primates (Preston and Whitlock, 1963; Muir and Porter, 1973; Shapovalov and Kurchavyi, 1974; Jankowska et al., 1975; Asanuma et al., 1979; Edgley et al., 1997). Preston and Whitlock (1963) and Uemura and Preston (1965), studying monosynaptic reflex conditioning in the “pyramidal” monkey preparation, in which the brainstem is destroyed leaving only the pyramidal tract intact, reported corticospinal output to soleus motoneurons was predominantly inhibitory while output to motoneurons of fast muscles (gastrocnemius and TA) was excitatory. Jankowska et al. (1975) reported EPSPs in soleus, but found the EPSPs in soleus and gastrocnemius were half the size of EPSPs in TA in the monkey. Kawai (1982), in the “pyramidal” cat preparation, demonstrated largely excitatory postsynaptic potentials (EPSPs) to fast motoneurons and inhibitory postsynaptic potentials (IPSPs) to slow motoneurons. Also in the cat, Binder et al. (1998) measured effective synaptic currents in fast and slow motoneurons of triceps surae associated with stimulating the contralateral pyramidal tract. They reported that more than 60% of putative slow motoneurons received a net hyperpolarizing effective synaptic current from pyramidal tract stimulation compared to only 33% of fast motoneurons. Consistent with this result, they also found that pyramidal tract stimulation increased the discharge rate of motoneurons receiving depolarizing effective currents while decreasing the rate of those receiving hyperpolarizing currents.

Transcranial magnetic stimulation (TMS) and transcranial electrical stimulation (TES) have been used in numerous human subject studies yielding varied results. TMS of motor cortex in humans consistently reveals a clear, short latency facilitation of the ankle flexor, TA (Brouwer and Ashby, 1990, 1992; Valls-Solé et al., 1994; Brouwer and Qiao, 1995; Ertekin et al., 1995; Goulart and Valls-Solé, 2001; Bawa et al., 2002; Geertsen et al., 2010). However, the results for the slow ankle extensor, soleus, have been more varied. Several studies have reported either non-existent or weak facilitation of soleus from TMS or electrical stimulation of the cortex (Cowan et al., 1986; Ashby and Advani, 1990; Brouwer and Ashby, 1990, 1992; Brouwer and Qiao, 1995), while other studies have shown that TMS does yield short latency facilitation of soleus (Valls-Solé et al., 1994; Goulart and Valls-Solé, 2001; Bawa et al., 2002; Geertsen et al., 2010).

Despite numerous studies in animals and humans, the cortical control of fast and slow muscles of the ankle remains controversial. The goal of this study was to investigate the cortical control of fast and slow muscles of the ankle in the rhesus macaque using stimulus triggered averaging (StTA) of EMG activity recorded from TA, MG, LG, and soleus (SOL) muscles during a hindlimb push-pull task. StTAing of EMG activity is a potentially more sensitive and higher resolution approach to delineating cortical motor output effects on muscle activity than the methods applied in previous studies. Also, unlike intracellular recording, it can be applied in awake, behaviorally active subjects thus avoiding the complicating effects of anesthesia or central lesions used with intracellular recording studies.

## Materials and methods

### Behavioral task

**Figure 1:**
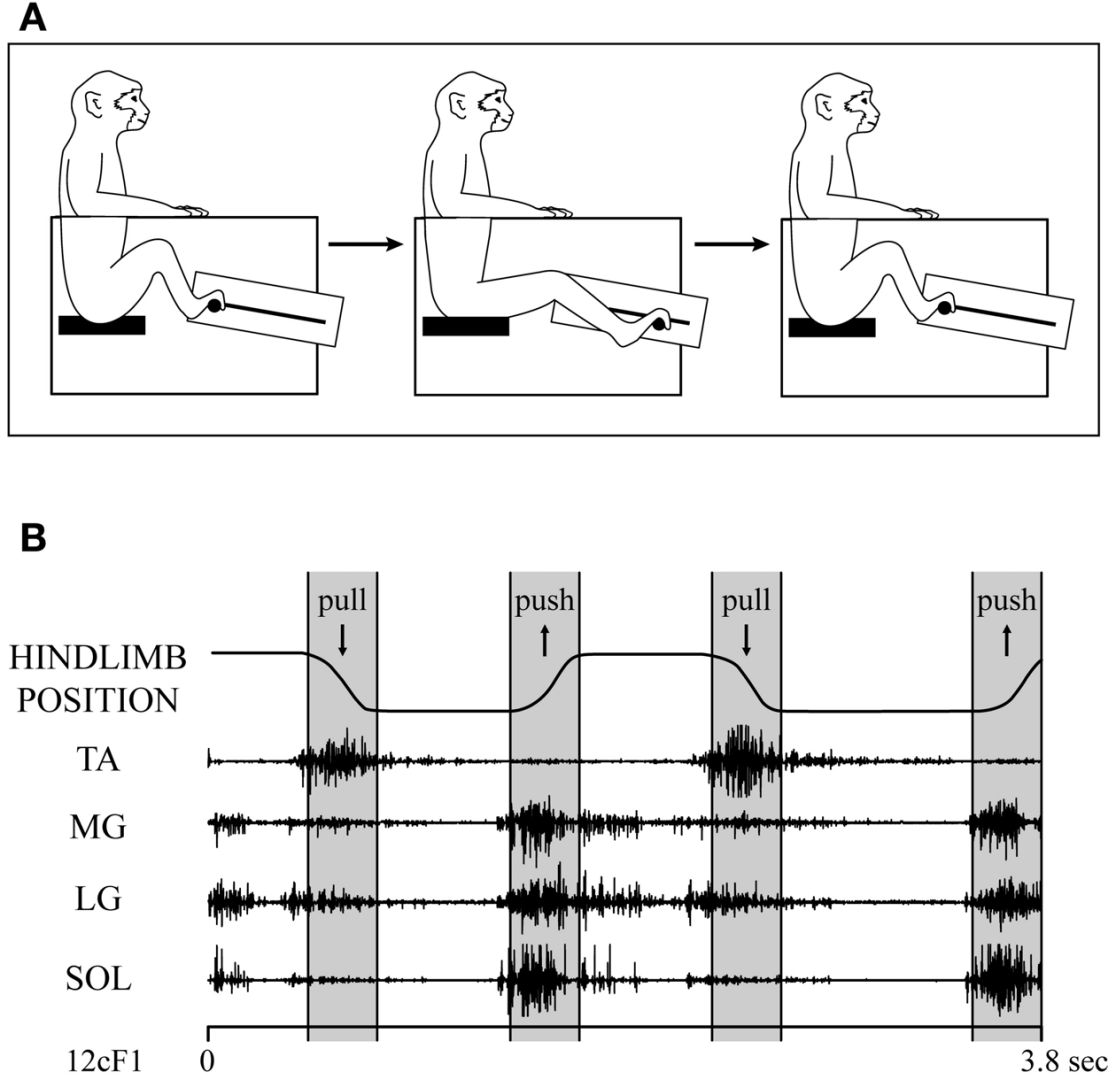
**(A)** Hindlimb push-pull task. The monkey is seated in a custom-built primate chair with both arms and the left leg restrained. The monkey gripped the push-pull device's manipulandum (horizontal post) with the right foot and pushed it to a target zone. After a hold period of 500 ms in the target zone, the monkey pulled the manipulandum to a second target zone and held for 500 ms. Upon successful completion of each push-pull trial, the monkey was given an applesauce reward. The behavioral task was guided by visual and auditory cues. **(B)** EMG records of ankle muscles during two cycles of the hindlimb push-pull task. TA, tibialis anterior; MG, medial gastrocnemius; LG, lateral gastrocnemius; SOL, soleus. Figure adapted from Hudson et al. (2010).

Data were collected from the left primary motor cortex (M1) of two male rhesus macaques (*Macaca mulatta*, ~10 kg, 6–7 years old). The monkeys were trained to perform a hindlimb push-pull task (Figure 1A) engaging both proximal and distal muscles in reliable and stereotyped patterns of activation (Hudson et al., 2010). Seated in a custom primate chair within a sound-attenuating chamber, both arms and the left leg were restrained. With the right foot, the monkey gripped the manipulandum (horizontal post) and extended the leg until the target zone was achieved. After a hold period of 500 ms in the target zone, the monkey flexed the leg pulling the manipulandum to a second target zone. Following a second hold period of 500 ms, the monkey was given an applesauce reward. The behavioral task was guided by visual and auditory cues.

### MRI

The monkey's head was placed in an MRI-compatible stereotaxic apparatus and structural MRIs in the sagittal, coronal and horizontal planes were obtained using a Siemens Allegra 3T system. A 3-dimensional reconstruction of each monkey's brain was produced using CARET software (Computerized Anatomical Reconstruction and Editing Tool Kit). This enabled highly accurate targeting of the hindlimb representation of M1 for the cortical chamber implant.

### Surgical procedures

Upon completion of training, each monkey was implanted with a titanium cortical recording chamber (30 mm inside diameter) centered at anterior 13.5 mm, lateral 0 mm and 0° angle to the midsagittal plane (Paxinos et al., 2000), targeting the hindlimb representation of M1. In a second surgery, pairs of insulated, multi-stranded stainless steel wire (Cooner Wire, AS632) were implanted in 19 muscles of the right hindlimb (Hudson et al., 2010). Briefly, pairs of wires were tunneled subcutaneously to their target muscles from either an external circular connector (Amphenol) affixed to the skull using dental acrylic and titanium screws (cranial-mounted subcutaneous implant, monkey C) or four external connector modules (ITT Canon) affixed to the upper arm with elastic medical adhesive tape (arm-mounted subcutaneous implant, monkey F). Proper placement of electrode pairs in each muscle was tested by stimulating through the electrodes with brief stimulus trains (biphasic pulse, 0.2 ms/phase, ~50 Hz) while observing appropriate evoked movements. Wires were removed and reinserted if proper placement was not confirmed. Similar stimulation tests were performed at various times after implantation to confirm electrode location. Within weeks of implantation, loops of extra wire length tucked into a subcutaneous pocket in the back became embedded in connective tissue rigidly anchoring the electrodes in place. While 19 hindlimb muscles were implanted in each monkey, this paper focuses on the results of EMGs recorded from four ankle muscles: TA, medial gastrocnemius (MG), lateral gastrocnemius (LG) and soleus (SOL) (Figure 1B).

All procedures were in accordance with the standards outlined by the *Guide for the Care and Use of Laboratory Animals* published by the US Department of Health and Human Services and the National Institutes of Health. All surgeries were performed in an Association for Assessment and Accreditation of Laboratory Animal Care (AAALAC) accredited facility using full aseptic procedures. Postoperative analgesics (buprenorphine, 0.01 mg/kg) were administered for 5 days. Wound edges were inspected daily and treated with Betadine (10% povidone-iodine) and topical antibiotic when necessary.

### Data collection

EMG activity, cortical activity and task-related signals were simultaneously monitored. Glass and mylar-insulated platinum-iridium electrodes (0.5–1.5 MΩ impedances, Frederick Haer) were used to record cortical unit activity and for stimulation. The electrode was positioned in the recording chamber using a custom-made x–y positioner and advanced using a manual hydraulic microdrive (Frederick Haer). Electrode penetrations were systematically made at 1 mm intervals in the precentral cortex of the left hemisphere encompassing the entire hindlimb M1 representation. Data were collected from putative sites in layer V of the cortex, as determined by depth from first cortical activity and size and nature of neuronal spikes. Data were collected from putative layer V sites in the bank of the medial wall and central sulcus at 0.5 mm intervals over the extent of the electrode track.

### Data analysis

At each putative layer V site, stimulus triggered averages (StTAs) (15, 30, and 60 μA at 15 Hz) of EMG activity were computed for 19 muscles of the hindlimb as the monkey performed the push-pull task. Individual stimuli were symmetrical biphasic pulses, 0.2 ms negative pulse followed by a 0.2 ms positive pulse, applied throughout all phases of the task. EMGs were generally filtered at 30 Hz to 1 kHz, digitized at 4 kHz and full-wave rectified. StTAs were compiled over an 80 ms epoch, 20 ms pre-trigger and 60 ms post-trigger, and consisted of at least 500 trigger events. To prevent averaging periods where EMG activity was minimal or non-existent, segments of EMG activity associated with each stimulus were evaluated and accepted for averaging only when the average of all EMG data points over the entire 80 ms epoch was ≥5% of full-scale input (McKiernan et al., 1998).

**Figure 2:**
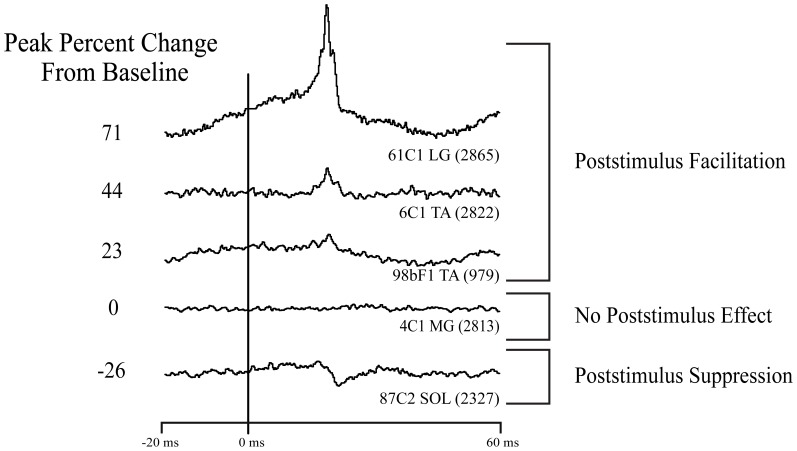
**Types of effects observed in stimulus triggered averages of ankle muscle EMG activity.** Column on left: magnitude of the primary poststimulus facilitation (PStF) or poststimulus suppression (PStS) measured as peak percent change from baseline EMG value just before the onset of the effect. Stimulation at 60 μA and 15 Hz repetition rate.

For this study, we analyzed StTAs from the four ankle muscles at each cortical site. Poststimulus facilitation (PStF) and suppression (PStS) effects were computer measured as described by Mewes and Cheney (1991). Each average consisted of an 80 ms epoch, 20 ms pre-trigger and 60 ms post-trigger. A poststimulus effect (PStE) was defined as a peak or trough of EMG activity that rose or fell from baseline and maintained a level exceeding two standard deviations of baseline for a period equal to or greater than 0.75 ms. Baseline EMG activity was measured as the 12 ms period preceding the onset of the effect initially determined by visual inspection. Baseline statistics were then used to determine the onset of the effect as the point where the envelope of the record exceeded two standard deviations of baseline. The magnitude of PStEs was expressed as the peak percentage increase (+ppi) or peak percentage decrease (−ppi) in EMG activity above (PStF) or below (PStS) baseline. To avoid skewing of the data from very weak effects, only PStF effects with a ppi ≥15 and PStS effects with a ppi ≤−15 were included in the analysis (Figure 2).

Cross-talk between muscles was evaluated by computing EMG-triggered averages (Cheney and Fetz, 1980). Averages of EMG activity were compiled for each muscle using one muscle's EMG activity as a trigger and repeated using all 19 muscles as triggers. If the ratio of the cross-talk peak in the test versus trigger muscle exceeded the ratio of their PStEs by a factor of two or more, a muscle was considered to have an unacceptable level of cross-talk (Buys et al., 1986). No muscle in this study showed significant cross-talk using this criterion.

### Unfolding the cortex

A two-dimensional representation of cortical layer V of the cortex in the medial wall of the hemisphere, the anterior bank of the central sulcus and the surface cortex required flattening and unfolding the curvature of the cortex. This process has been described in detail by Park et al. (2001). Briefly, the cortex was unfolded and 2-dimensional maps were generated based on known architectural landmarks, observations during the cortical chamber implant surgery, MRI images, electrode track x-y coordinates, electrode penetration depth and properties of recorded neurons.

## Results

### EMG activity during behavioral task

Figure 1B shows the EMG activity of TA, MG, LG, and SOL throughout different phases of the hindlimb task. The extensors (MG, LG, and SOL) all showed a similar pattern of modulation with the strongest activity during the extension (push) phase of the task but continuing at a lower level through the hold phase of extension and also at a lower level yet during leg flexion (pull). The flexor muscle (TA) showed a more focused pattern with activity confined primarily to the flexion (pull and hold) phase of the task.

### Dataset

StTA of EMG activity from four ankle muscles was performed systematically from sites in the left M1 cortex of two rhesus macaques. Figure 2 illustrates the types of PStEs obtained (facilitation, suppression, no effect). A total of 312 electrode tracks were made (monkey F, 170; monkey C, 142). Data collection is summarized in Table 1. StTA was performed at 259 putative layer V sites at 15 μA, 292 sites at 30 μA and 317 sites at 60 μA. Twenty-seven putative layer V sites yielded PStEs at 15 μA, 73 at 30 μA and 134 at 60 μA. Both PStF and PStS were observed in each of the four ankle muscles. Data from all sites were used to analyze the distribution of effects (Table 2A). Data from sites with PStEs in the same muscle at all three stimulus intensities are shown separately (Table 2B). Although the number of effects is somewhat limited, these data provide a purer measure of changes in magnitude with stimulus intensity.

**Table 1:** **Summary of data collected from ankle muscles**.

	**Monkey F**			**Monkey C**			**Total**		
**Electrode tracks**	**170**			**142**			**312**		
	**15 μA**	**30 μA**	**60 μA**	**15 μA**	**30 μA**	**60 μA**	**15 μA**	**30 μA**	**60 μA**
Layer V^*^ sites stimulated	117	150	167	142	142	150	259	292	317
Sites yielding PStEs	4	17	55	23	56	79	27	73	134
Sites yielding PStF	4	15	47	14	35	66	18	50	113
Sites yielding PStS	0	2	11	11	22	29	11	24	40
PStEs obtained	4	20	99	29	89	179	33	109	278
PStF effects	4	18	84	17	61	136	21	79	220
PStS effects	0	2	15	12	28	43	12	30	58

PStE, poststimulus effect; PStF, poststimulus facilitation; PStS, poststimulus suppression.

* Putative layer V sites identified based on criteria given in the text.

**Table 2:** **Latency and magnitude of PStEs**.

**A. All effects**												
	**Onset latency, ms**						**Magnitude, %**					
	**15 μA**		**30 μA**		**60 μA**		**15 μA**		**30 μA**		**60 μA**	
**Muscle**	***n***	**Mean**	***n***	**Mean**	***n***	**Mean**	***n***	**Mean**	***n***	**Mean**	***n***	**Mean**
**PStF**												
TA	6	20.3 ± 3.3	18	16.3 ± 3.2	54	17.3 ± 3.9	6	23.7 ± 4.3	18	26.2 ± 10.0	54	29.9 ± 10.5
SOL	7	19.3 ± 3.1	21	16.9 ± 1.9	68	16.6 ± 2.0	7	24.6 ± 10.9	21	25.6 ± 8.2	68	30.9 ± 14.7
LG	3	16.3 ± 0.9	29	16.2 ± 1.1	65	15.6 ± 1.3	3	19.0 ± 0.7	29	21.8 ± 4.8	65	33.4 ± 16.3
MG	5	16.5 ± 1.4	11	17.6 ± 3.4	33	15.8 ± 2.4	5	28.2 ± 11.6	11	28.9 ± 18.8	33	24.2 ± 8.6
Total	21	18.5 ± 3.0	79	16.6 ± 2.3	220	16.3 ± 2.6	21	24.4 ± 8.7	79	24.8 ± 9.9	220	30.4 ± 13.8
**PStS**												
TA	3	24.9 ± 1.1	7	21.9 ± 3.3	17	23.2 ± 2.8	3	−19.9 ± 3.6	7	−17.8 ± 2.2	17	−22.2 ± 5.5
SOL	7	18.6 ± 3.1	15	17.6 ± 2.1	22	18.3 ± 1.4	7	−19.4 ± 3.8	15	−24.3 ± 7.6	22	−24.2 ± 7.8
LG	1	17.8	4	18.6 ± 0.8	8	19.0 ± 0.8	1	−15.7	4	−19.3 ± 3.5	8	−22.2 ± 5.2
MG	1	16.8	4	18.3 ± 2.1	11	19.0 ± 2.4	1	−16.6	4	−18.3 ± 2.4	11	−20.3 ± 5.1
Total	12	19.9 ± 3.8	30	18.8 ± 2.8	58	20.0 ± 2.9	12	−19.0 ± 3.6	30	−21.3 ± 6.3	58	−22.6 ± 6.4
**B. Effects present in the same muscle at 15, 30, and 60 μA**												
**PStF**												
TA	5	19.5 ± 2.9	5	18.8 ± 3.0	5	19.2 ± 3.0	5	24.5 ± 4.3	5	34.8 ± 15.8	5	41.9 ± 5.6
SOL	3	17.9 ± 2.0	3	17.8 ± 2.0	3	18.2 ± 2.3	3	24.2 ± 10.9	3	31.9 ± 11.7	3	57.3 ± 30.1
LG	3	16.3 ± 0.9	3	16.9 ± 0.1	3	15.9 ± 1.0	3	19.0 ± 0.7	3	29.5 ± 4.3	3	51.0 ± 14.4
MG	1	17.5	1	17.8	1	17.0	1	20.8	1	31.8	1	27.8
Total	12	18.1 ± 2.4	12	18.0 ± 2.2	12	17.9 ± 2.5	12	22.8 ± 5.9	12	32.5 ± 11.1	12	46.8 ± 17.1
**PStS**												
TA	0	–	0	–	0	–	0	–	0	–	0	–
SOL	5	17.2 ± 1.4	5	17.5 ± 1.4	5	17.5 ± 1.2	5	−18.0 ± 1.6	5	−25.9 ± 7.4	5	−33.5 ± 9.9
LG	1	17.8	1	17.8	1	17.8	1	−15.7	1	−16.3	1	−26.0
MG	0	–	0	–	0	–	0	–	0	–	0	–
Total	6	17.3 ± 1.3	6	17.5 ± 1.3	6	17.5 ± 1.1	6	−17.6 ± 1.7	6	−24.3 ± 7.7	6	−32.3 ± 9.4

Values are mean ± SD. %, peak percent change from baseline; PStE, poststimulus effect; PStF, poststimulus facilitation; PStS, poststimulus suppression.

### Latency and magnitude

At 15 μA, the average PStF onset latency across all ankle muscles was 18.5 ± 3.0 ms compared with an average PStS onset latency of 19.9 ± 3.8 ms (Table 2A). The latency difference between PStF and PStS increased to 2.2 ms at 30 μA and 3.7 ms at 60 μA. There were no significant differences in PStF onset latency between muscles at any stimulus intensity (Kruskal–Wallis test, n.s.). As expected, the PStF onset latency decreased with stimulus intensity and this difference became statistically significant in some cases (TA at 15 μA compared to 30 and 60 μA; SOL at 15 μA compared to 60 μA; Wilcoxon signed ranks test, *p* < 0.05). For a given muscle, PStS onset latency was not different at any stimulus intensity although the numbers of effects for MG and LG at 15 μA were too small for comparison (Table 2A, Friedman's test, n.s.) At 60 μA, TA PStS onset latency was greater than MG, LG, and SOL (Mann–Whitney's U test, TA-MG *p* = 0.001, TA-LG *p* < 0.05, TA-SOL *p* < 0.001).

**Figure 3:**
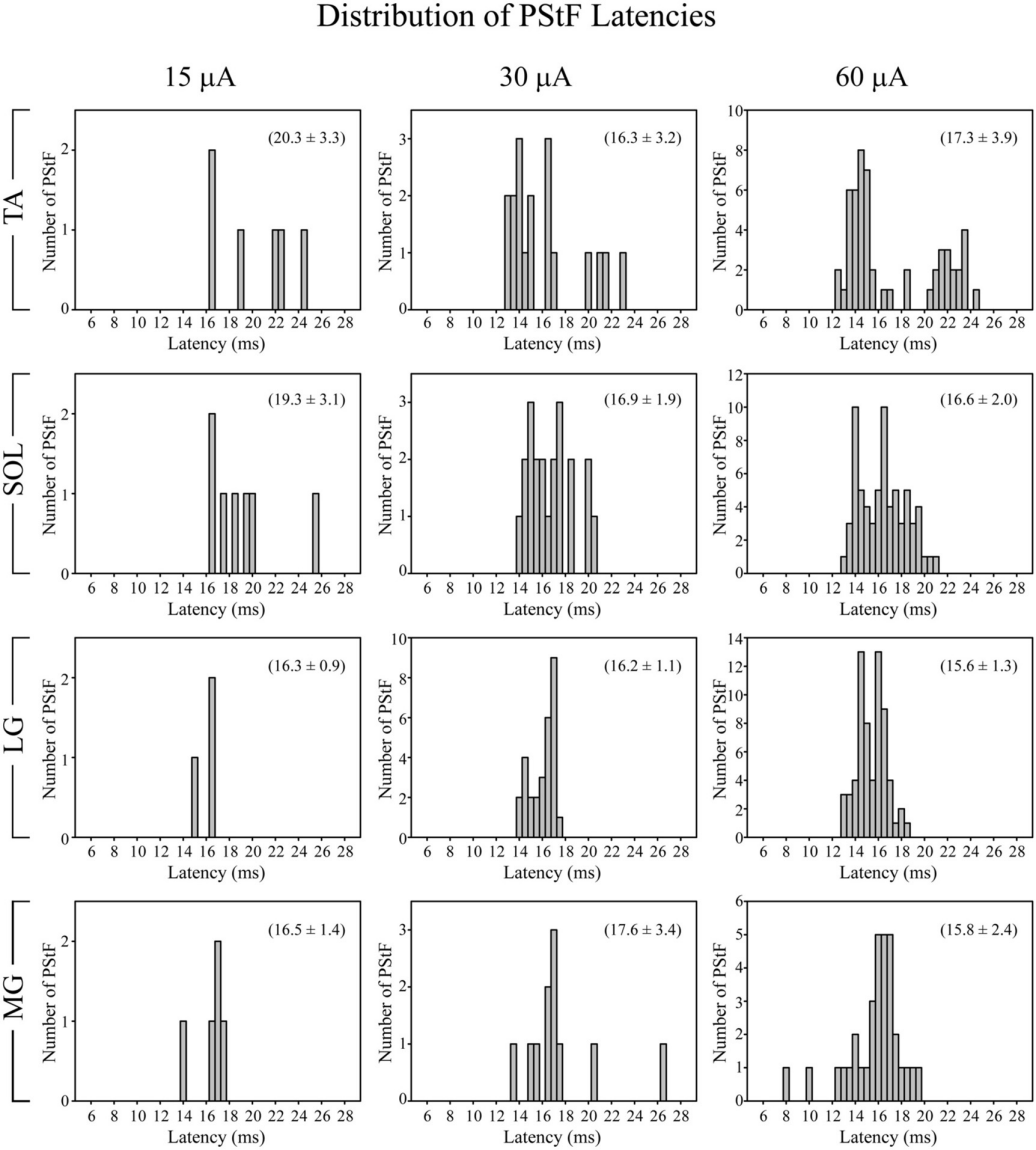
**Distribution of PStF onset latencies for ankle muscles at 15, 30, and 60 μA stimuli.** The values given in parentheses for each graph represent the mean ± SD of the onset latency of the PStF. Muscle abbreviations are the same as in Figure 1.

Figure 3 shows the distribution of PStF onset latencies for the ankle muscles at 15, 30, and 60 μA (all effects included). There was a similar distribution of latencies among all muscles, although TA had a clear suggestion of bimodality that was not present in the distributions for other muscles. The minimum onset latency of PStF decreased by 1.9 ms from 15 to 30 μA and by 0.3 ms from 30 to 60 μA (Table 2A). Regardless of muscle, the minimum latency was approximately 12–13 ms (30 and 60 μA). The only exceptions were two effects in MG at 60 μA that were 8 and 10 ms.

At 15 μA, the overall mean PStF magnitude, expressed as peak percent increase (ppi) above baseline, was 24.4 ± 8.7 compared with a peak percent decrease of −19.0 ± 3.6 for PStS (Table 2A). When comparing mean PStF magnitude across muscles, there were no significant differences between muscles at 15 and 30 μA (Kruskal–Wallis test, n.s.). However, at 60 μA MG had a significantly weaker PStF magnitude than LG, TA, and SOL (Mann–Whitney's U test, *p* < 0.01). There were no significant differences between muscles for PStS magnitude at any stimulus intensity (Kruskal–Wallis test, n.s.). Changes in magnitude of effects with stimulus intensity are best appreciated from a subset of cortical sites in which effects were present at each of the three stimulus intensities (Table 2B). Although the number of sites is somewhat limited, the data show increases in PStF magnitude ranging from 7.7–11.0% in going from 15 to 30 μA and 7.1–25.4% in going from 30 to 60 μA. Corresponding increases for PStS were 0.6–7.9% for 15–30 μA and 7.6–9.7% for 30–60 μA. Magnitudes based on all effects (Table 2A) are not appropriate for examining relationships between magnitude and intensity because higher intensity stimulation recruits new muscles with weak effects that dilute the mean magnitude.

**Figure 4:**
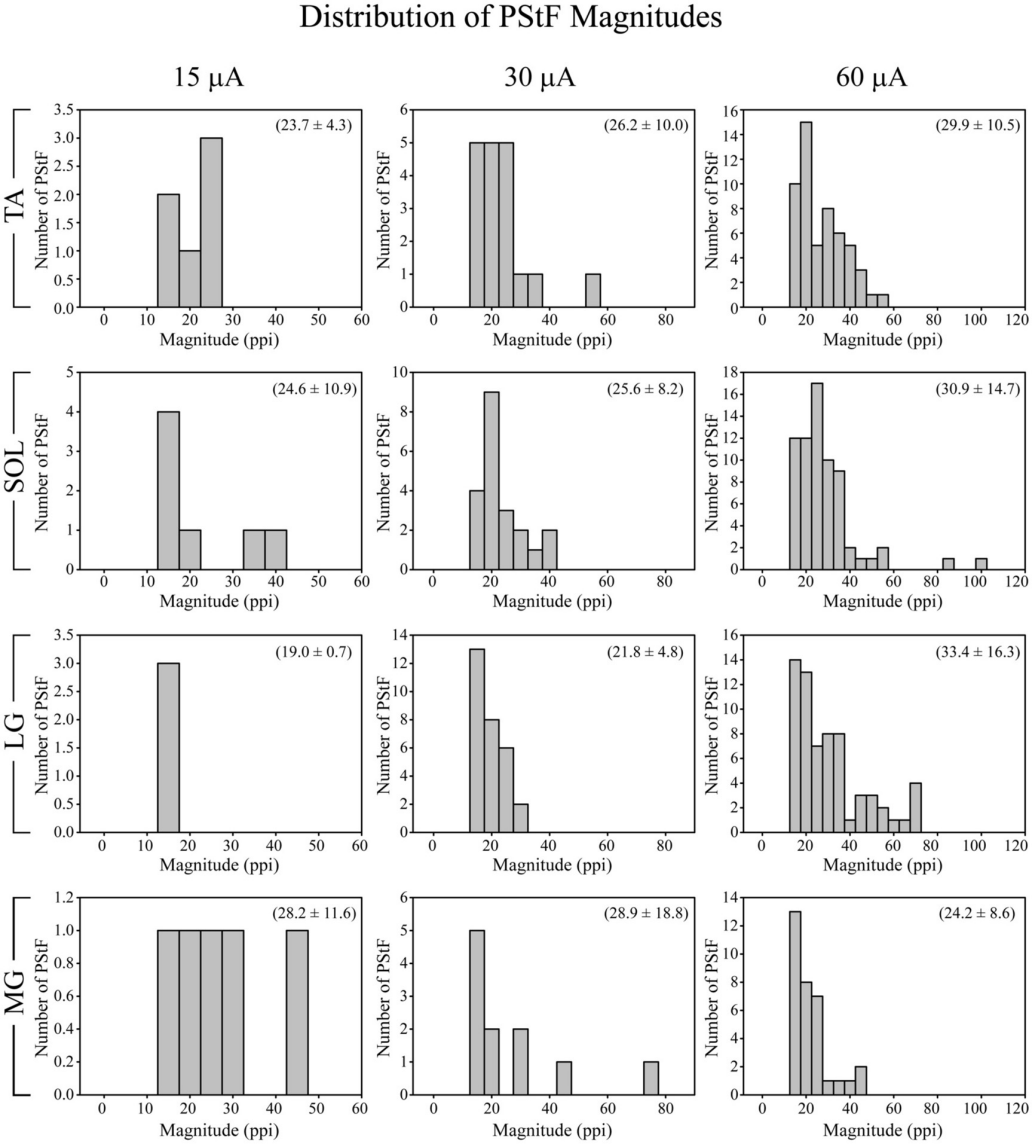
**Distribution of PStF magnitudes for ankle muscles at 15, 30, and 60 μA stimuli.** The magnitudes are expressed as peak percent increase (ppi) above baseline. The values given in parentheses for each graph represent the mean ± SD of the magnitude of the PStF. Muscle abbreviations are the same as in Figure 1.

There was a similar distribution of magnitudes of PStF among all muscles with a consistent trend toward skewing in the direction of the weakest magnitudes (Figure 4), a trend also observed in the primate forelimb (Park et al., 2004). The strongest effects observed for each muscle were in the range of 60–70 ppi (60 μA). Effects in soleus were equally as strong as those in TA, MG, and LG. In fact, the two strongest effects observed were in soleus.

### Distribution of PStEs

**Figure 5:**
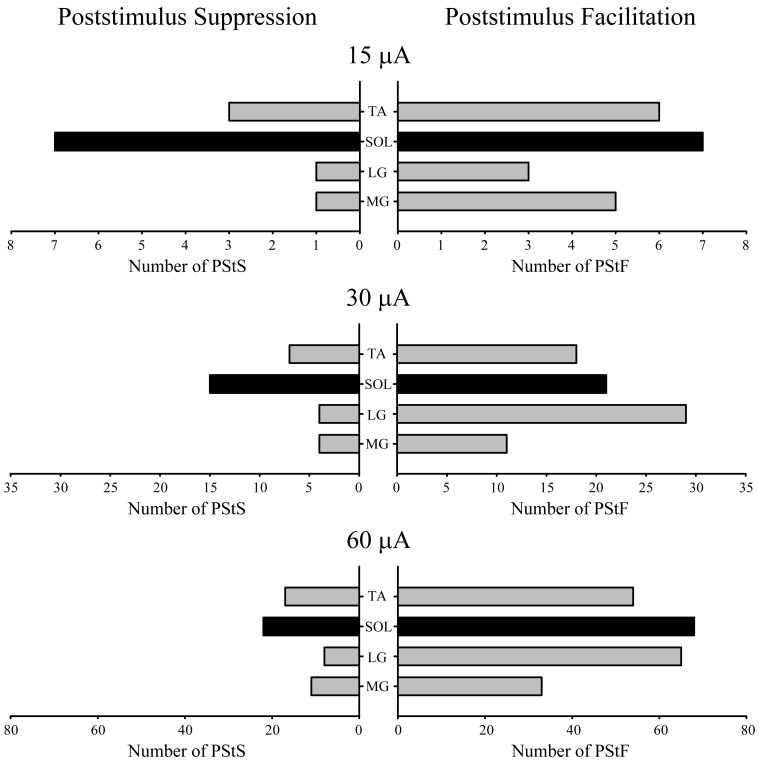
**Distribution of PStF (right) and PStS (left) effects obtained from four ankle muscles of the hindlimb at 15, 30, and 60 μA stimuli.** Gray bars: fast muscles. Black bars: slow muscle. Muscle abbreviations are the same as in Figure 1.

Figure 5 shows the distribution of PStF and PStS effects observed in each of the ankle muscles sampled at 15, 30, and 60 μA. Both PStF and PStS effects were observed in each muscle at each stimulus intensity. Overall, PStF was more common than PStS in all four muscles. PStS was most common in SOL, especially at 15 μA where the incidence of PStS was equal to the incidence of PStF. At higher intensities the incidence of PStF compared to PStS in SOL shifted in favor of PStF. Both monkeys exhibited these trends. However, it should be noted that the increased incidence of facilitation with increasing stimulus intensity is likely to be a consequence of the fact that, for clarity, we based the sign of an effect (facilitation or suppression) on the earliest latency component. Because output zones in cortex are mixed and PStF has a shorter latency than PStS, as stimulus intensity increases, changes in the incidence of facilitation and suppression will be biased toward facilitation. Accordingly, results at the 15 μA intensity are likely to be most meaningful relative to questions about the prevalence of facilitation versus suppression in different muscles.

Suggestions in previous studies of differential control of fast and slow muscles from motor cortex (Preston and Whitlock, 1963) prompted us to examine this issue with our data. It was proposed that cortically initiated movements could be enhanced through cortical inhibition of soleus as a slow, tonically contracting postural muscle, coupled with excitation of its agonists—MG and LG. We examined this issue by determining the relative prevalence of PStS and PStF in the gastrocnemius muscles and TA when (1) PStS was present in soleus, and (2) PStF was present in soleus. The results show that in all cases and at all intensities, the effect in soleus tends to be matched by a similar effect in MG and LG. For instance, at 60 μA, there were 22 PStS effects in soleus. In these cases, there was one PStF effect in the gastrocnemius muscles and 15 PStS effects. The opposite pattern was evident for TA which followed a reciprocal innervation plan with 8 PStF effects and 2 PStS effects.

### Muscle representation

**Figure 6:**
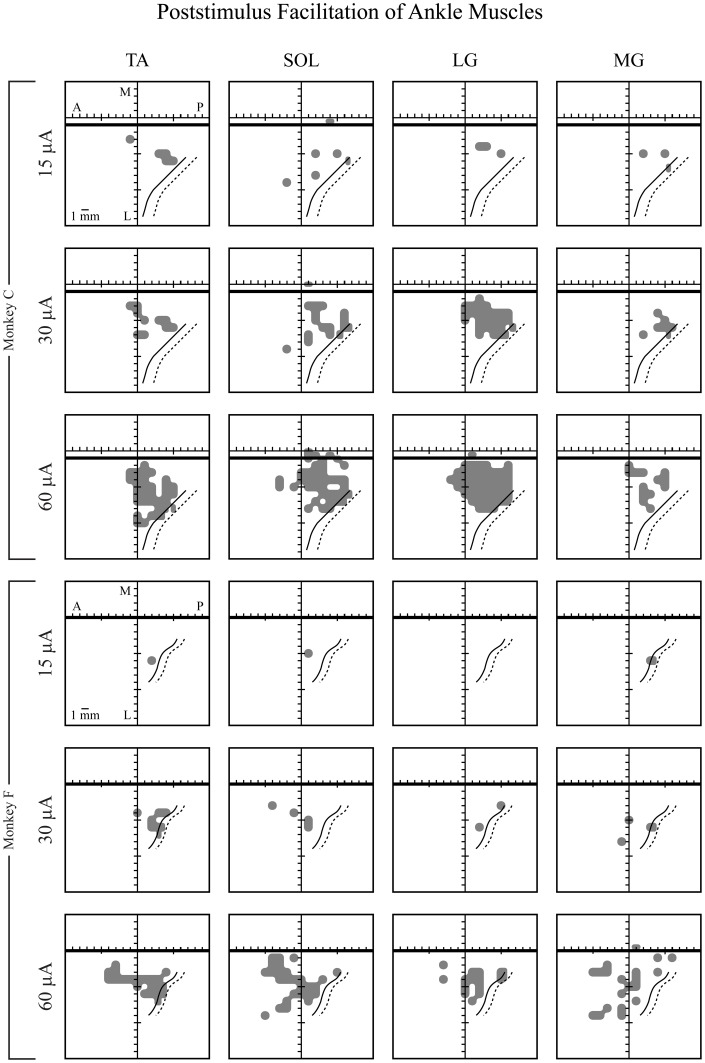
**Maps of individual ankle muscle representations in hindlimb primary motor cortex in two monkeys (F and C), represented in two dimensions after unfolding the medial wall and central sulcus.** Maps of hindlimb muscles were based on PStF effects at 15, 30, and 60 μA. Heavy horizontal black line: midline, above the heavy black line represents the bank of the medial wall of the hemisphere. Solid black curved line: central sulcus. Dotted black curved line: fundus of the central sulcus. A, anterior; P, posterior; M, medial; and L, lateral. Muscle abbreviations are the same as in Figure 1.

Figure 6 shows the representation of each muscle in M1 of both monkeys based on PStF effects. All muscles were represented in both monkeys. There was massive overlap in the territories for each muscle, not only of the extensors (MG, LG, and SOL) but also of the flexor muscle, TA. While monkey F had considerably fewer effects than monkey C, the same trends were apparent in both monkeys.

## Discussion

Early studies in the primate and more recent studies in human subjects have yielded conflicting results regarding the role of motor cortex in the control of slow muscles such as soleus. While it is well established that the motor cortex has a dominant excitatory effect on fast muscles, the cortical control of the slow muscle, soleus, has remained contentious. In this study, we investigated the cortical control of fast (TA, MG, and LG) and slow (SOL) muscles of the ankle using StTA of EMG activity (Cheney and Fetz, 1985; Park et al., 2001). With this method, microstimuli are superimposed on a background of EMG activity associated with task performance. The effects of single stimuli are subthreshold for overt EMG responses but the evoked EPSPs and IPSPs in motoneurons influence the firing probability of motoneurons and this can be revealed with signal averaging of EMG over thousands of stimuli. This provides a highly sensitive method capable of revealing both excitatory and inhibitory effects.

PStF was observed in all muscles and was as common in the slow muscle, soleus, as in the fast muscles. The mean onset latencies of PStF effects were similar among the fast and slow muscles. The mean magnitudes of PStF effects were also similar among the fast and slow muscles. The distributions of PStF magnitudes were similar among all muscles, demonstrating a consistent trend toward the weakest magnitudes being the most common. Poststimulus suppression (PStS) was observed in all muscles; however, it was more common in soleus than in the fast muscles, especially at lower stimulus intensities where it was as common as facilitation. This was not true of fast muscles at any stimulus intensity.

The question arises as to why previous studies in the primate and some studies in human subjects have reported weak to absent excitatory effects on soleus or predominantly inhibitory effects from motor cortex in contrast to our own results. The early studies of Preston and Whitlock (1963) and Uemura and Preston (1965) used the monkey “pyramidal” preparation, which involves lesioning the brainstem sparing only the pyramidal tract, while the monkeys in our study were awake, with an intact nervous system, and performing a trained motor behavioral task. It should be noted that while Preston and Whitlock (1963) emphasized the predominant, almost pure inhibitory, nature of the effect of cortical stimulation on motoneurons of soleus determined with monosynaptic reflex conditioning, early weak facilitation peaks were evident but considered equivocal (their Figure 3). They characterized their results as showing “quantitatively greater cortical inhibition impinging on soleus motoneurons when compared with the synergistic motoneurons of the medial head of the gastrocnemius muscle” and this statement is consistent with our results.

In human subjects, Bawa et al. (2002) found that a higher stimulus intensity was needed to elicit a short latency facilitation in soleus compared to TA. Moreover, Valls-Solé et al. (1994) found that TMS effects in soleus were difficult to elicit unless the subjects were standing on their toes which would produce a large excitability increase in soleus motoneurons. Consistent with this finding, Goulart and Valls-Solé (2001) reported that facilitation of soleus was stronger when subjects were in standing position rather than seated. Geertsen et al. (2010) found that facilitation of soleus occurred during voluntary ankle extension and flexion. Reports of weak or non-existent soleus excitation may have been due to low stimulus intensities (Brouwer and Qiao, 1995) or inadequate background excitability of soleus motoneurons. In this study, we used three stimulus intensities and found the relative number of suppression and facilitation effects in soleus to be similar at low intensities (15 μA). At higher intensities, facilitation of soleus was more common than suppression.

The results obtained in this study for hindlimb muscles are of interest in comparison with results obtained with similar methods for forelimb muscles. Park et al. (2004) examined M1 output effects in 24 muscles of the macaque forelimb using 15 μA StTA. Although there are no pure slow muscles acting at the wrist for comparison with our data on soleus, it is possible to compare effects on ankle muscles obtained in this study with those for wrist muscles from Park et al., 2004. In general, wrist PStF effects were considerably greater in magnitude compared to ankle PStF effects. At 15 μA, the average wrist PStF magnitude (ppi) was 73.7 ± 81.1 compared to an ankle muscle average PStF magnitude of 24.4 ± 8.7. PStS showed a similar pattern, although the disparity was not as great. At 15 μA, the average wrist PStS magnitude was −30.8 ± 15.2 compared to an ankle muscle average PStS magnitude of −19.0 ± 3.6. The magnitude of ankle muscle PStS was 78% of that for PStF, whereas for wrist muscles it was only 42%. As for the distribution of excitatory and inhibitory effects, PStF was more common than PStS in all wrist muscles at 15 μA (77 vs. 33%). Other intensities were not tested. The same was true of ankle muscles at 15 μA except soleus, where PStF and PStS were equally common. At higher stimulus intensities, facilitation in soleus was more common than suppression. However, this would be expected from the mixed cortical representations of facilitation and suppression coupled with the fact that facilitation effects are shorter latency and we used the shortest latency effect to define the sign (facilitation or suppression) of the effect.

Based on our results, there can be no doubt that motor cortex in primates is capable of powerful excitatory effects on soleus motoneurons equal to that of fast muscles. However, it should be emphasized that motor cortex is also capable of significant inhibitory effects on soleus and the inhibitory effects are more prominent in the slow muscle, soleus, than the fast muscles. In fact, at low stimulus intensities, inhibitory effects in soleus were as common as excitatory effects. Overall, our results support the findings of recent TMS studies in human subjects demonstrating short latency facilitation of both fast and slow muscles of the ankle (Valls-Solé et al., 1994; Goulart and Valls-Solé, 2001; Bawa et al., 2002; Geertsen et al., 2010), but also leave open the possibility for a unique role of cortical inhibition of soleus in the control of movement.

### Conflict of interest statement

The authors declare that the research was conducted in the absence of any commercial or financial relationships that could be construed as a potential conflict of interest.
